# Flying between raindrops: Strong seasonal turnover of several Lepidoptera groups in lowland rainforests of Mount Cameroon

**DOI:** 10.1002/ece3.4704

**Published:** 2018-12-03

**Authors:** Vincent Maicher, Szabolcs Sáfián, Mercy Murkwe, Łukasz Przybyłowicz, Štěpán Janeček, Eric B. Fokam, Tomasz Pyrcz, Robert Tropek

**Affiliations:** ^1^ Institute of Entomology, Biology Centre Czech Academy of Sciences Ceske Budejovice Czech Republic; ^2^ Faculty of Science University of South Bohemia Ceske Budejovice Czech Republic; ^3^ Institute of Silviculture and Forest Protection, Faculty of Forestry University of West Hungary Sopron Hungary; ^4^ Department of Zoology and Animal Physiology, Faculty of Science University of Buea Buea Cameroon; ^5^ Department of Ecology, Faculty of Science Charles University Prague Czech Republic; ^6^ Institute of Systematics and Evolution of Animals Polish Academy of Sciences Krakow Poland; ^7^ Institute of Botany Czech Academy of Sciences Trebon Czech Republic; ^8^ Institute of Zoology and Biomedical Research Jagiellonian University Krakow Poland; ^9^ Nature Education Centre Jagiellonian University Krakow Poland

**Keywords:** Afrotropics, biodiversity patterns, Lepidoptera, multitaxa approach, phenology, seasonality

## Abstract

Although seasonality in the tropics is often less pronounced than in temperate areas, tropical ecosystems show seasonal dynamics as well. Nevertheless, individual tropical insects’ phenological patterns are still poorly understood, especially in the Afrotropics. To fill this gap, we investigated biodiversity patterns of Lepidoptera communities at three rainforest localities in the foothills of Mount Cameroon, West Africa, one of the wettest places in the world. Our multitaxa approach covered six lepidopteran groups (fruit‐feeding butterflies and moths, the families Sphingidae, Saturniidae, and Eupterotidae, and the subfamily Arctiinae of Erebidae) with diverse life strategies. We sampled adults of the focal groups in three distinct seasons. Our sampling included standardized bait trapping (80 traps exposed for 10 days per locality and season) and attraction by light (six full nights per locality and season). Altogether, our dataset comprised 20,576 specimens belonging to 559 (morpho)species of the focal groups. The biodiversity of Lepidoptera generally increased in the high‐dry season, and either increased (fruit‐feeding moths, Arctiinae, Saturniidae) or decreased (butterflies, Sphingidae) in the transition to the wet season in particular groups. Simultaneously, we revealed a strong species turnover of fruit‐feeding Lepidoptera and Arctiinae among the seasons, indicating relatively high specialization of these communities for particular seasons. Such temporal specialization can make the local communities of butterflies and moths especially sensitive to the expected seasonal perturbations caused by the global change. Because of the key role of Lepidoptera across trophic levels, such changes in their communities could strengthen this impact on entire tropical ecosystems.

## 1 INTRODUCTION

Understanding the spatial and temporal dynamics of biodiversity is one of the main goals of current ecology (Magurran, 2007; Rosenzweig, 1995). Although spatial patterns of biodiversity have been widely studied, research on its temporal dynamics in natural conditions remains strongly challenging and thus much less common. This is especially valid for the tropics, where the seasonal biodiversity patterns still remain poorly understood (Kishimoto‐Yamada & Itioka, 2015).

In tropical rainforests, phenology of individual insect species, as well as of whole ecosystems, follows the regional seasonality typically represented by swapping of the wet and dry seasons (Kishimoto‐Yamada & Itioka, 2015; Wolda, 1988). Various tropical areas, usually with one or two annual rainy seasons, exhibit annual or biannual peaks of adult Lepidoptera species richness, as well as phenological patterns in their communities' composition (Cruz‐Neto, Machado, Duarte, & Lopes, 2011; DeVries, Murray, & Lande, 1997; DeVries, Alexander, Chacon, & Fordyce, 2012; Devries & Walla, 2001; Grøtan, Lande, Engen, Sæther, & DeVries, 2012; Grøtan, Lande, Chacon, & Devries, 2014; Hilt, Brehm, & Fiedler, 2007; Intachat, Holloway, & Staines, 2001; Valtonen et al., 2013). However, until lately, our knowledge on the phenology of tropical rainforests insects, including Lepidoptera (i.e., butterflies and moths), suffered from a lack of comprehensive studies. Available detailed studies described seasonal changes of some selected lepidopteran groups and proposed mainly weather conditions and host–plant availability as the main drivers of Lepidoptera phenology in tropical rainforests (e.g., Intachat et al., 2001; DeVries et al., 2012; Grøtan et al., 2012; Grøtan et al., 2014; Valtonen et al., 2013). Several of them detected the main peak of adult Lepidoptera abundances (Intachat et al., 2001) and species richness (Grøtan et al., 2014, 2012 ; Valtonen et al., 2013) with a time lag of two or three months after the beginning of the wet season. Regardless, both temperature and rainfall fluctuations were revealed to influence lepidopterans' abundances and species richness in both directions (Grøtan et al., 2014, 2012 ; Kishimoto‐Yamada & Itioka, 2015; Wolda, 1988). A decreasing day temperature and increasing precipitation in the early rainy season negatively affect adults' activity (Holyoak, Jarosik, & Novák, 1997; Ribeiro & Freitas, 2010), while strong rainfalls and high humidity increase the mortality of early life stages by increasing the activity of pathogens, or by direct disturbance of caterpillars in their host plants (Hill, Hamer, Dawood, Tangah, & Chey, 2003; Intachat et al., 2001). On the other hand, rainfalls often trigger sprouting of young leaves important for caterpillars, especially in their earliest developmental stages (Hill et al., 2003; Valtonen et al., 2013), whereas the wettest part of the year also coincides with a higher predation rate on caterpillars (Molleman, Remmel, & Sam, 2016). Similarly, mass flowering in the late rainy and high‐dry seasons was described to support the biodiversity of adult geometroid moths (Intachat et al., 2001) and Sphingidae (Cruz‐Neto et al., 2011).

Individual groups of tropical Lepidoptera can differ in their phenological patterns (Ribeiro, Prado, Brown, & Freitas, 2010). The highest species richness of particular tropical lepidopteran groups was detected in different seasons: high‐dry season for Sphingidae (Cruz‐Neto et al., 2011; Owen, 1969), geometrids (Hilt et al., 2007) and butterflies (Aduse‐Poku et al., 2012; DeVries et al., 2012; Grøtan et al., 2014, 2012 ; Ribeiro et al., 2010), transition from wet to dry seasons for butterflies (Valtonen et al., 2013), and wet season for butterflies (Checa, Rodriguez, Willmott, & Liger, 2014; DeVries et al., 1997; Devries & Walla, 2001). No specific seasonal patterns of species richness were revealed for Sphingidae (Beck & Linsenmair, 2006), Arctiinae (Hilt et al., 2007), butterflies (Larsen, Riley, & Cornes, 1979; Molleman, Kop, Brakefield, Vries, & Zwaan, 2006; Owen & Chanter, 1972), pyraloids (Fiedler & Schulze, 2004; Schulze & Fiedler, 2003), and macro‐heterocerans (Tikoca et al., 2016). An overwhelming majority of these group‐specific patterns came from single‐taxon studies carried out in different tropical localities or even areas, often with different seasonality. It is thus very difficult to separate the effects of biogeography and individual lepidopteran groups' phenology with the current knowledge.

Moreover, most of the tropical Lepidoptera seasonality studies originated from South and Central America and Southeast Asia. Publications on the temporal dynamics of Afrotropical Lepidoptera are still relatively scarce and mostly focused on butterflies only (e.g., Owen & Chanter, 1972; Larsen et al., 1979; Molleman et al., 2006; Namu, Githaiga, Kioko, Ndegwa, & Hauser, 2008; Aduse‐Poku et al., 2012; Valtonen et al., 2013; but see Owen, 1969; Axmacher, Kühne, & Vohland, 2008). Recently, it was predicted that the global change will strongly affect the seasonality of rainfall in the tropics during the next century, with expected strong changes in the amount of precipitation in equatorial Africa (Feng, Porporato, & Rodriguez‐Iturbe, 2013). Together with the expected shifts in the seasonality timing (Feng et al., 2013), the Afrotropical Lepidoptera communities' phenology ought to shift or completely change, with unpredictable consequent effects on the related trophic levels. To predict such changes, it is firstly necessary to identify the current seasonal patterns of communities.

Here, we bring a detailed study of several taxonomical groups of adult lepidopterans from three different seasons in the lower altitudes of Mount Cameroon, West Africa. We address the following questions: (a) How does the extreme seasonality affect species richness, abundance, and diversity of local lepidopteran communities? (b) Are there any phenological patterns in community compositions of individual lepidopteran groups? (c) Are the phenological patterns caused by interseasonal species turnover or community nestedness? (d) Are these phenological patterns consistent across a few unrelated lepidopteran groups?

**Figure 1:**
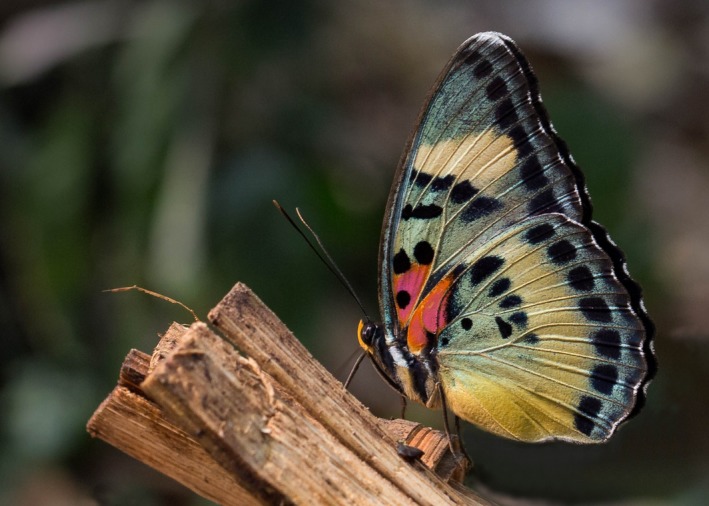
*Euphaedra permixtum* (Butler, 1873) is a fruit‐feeding butterfly typical for many tropical forests of West and Central Africa. Photo by Jan Mertens

To answer these questions, we combined an extensive standardized sampling by fruit‐baited traps with attraction by light. Considering the extreme seasonality within the study area, we expected continuously increasing biodiversity of individual focal groups after the extreme wet season, with the peak in the dry season for butterflies, and in the beginning of the wet season for moths. Simultaneously, we also expected relatively high interseasonal specialization of communities. Our study represents the first multitaxa survey of butterflies (Figure 1) and several moth groups using standardized sampling at light and by bait traps in the Afrotropical region. Simultaneously, our bait trapping is among the most intensive worldwide. Because Mount Cameroon is one of the rainiest regions in the world, with a strong discrepancy between high‐dry and high‐wet seasons (Proctor, Edwards, Payton, & Nagy, 2007), we expected distinct seasonal patterns of both species richness and community composition.

## 2 MATERIALS AND METHODS

### 2.1 Study area and sites

Mount Cameroon is the highest mountain in West and Central Africa, rising directly from the seashore to its peak at 4,095 m a.s.l. It is located in the southwestern part of the Cameroon Volcanic Line (also known as Gulf of Guinea Highland), being the only active volcano in the region. Its slopes, excluding the eastern one adjoining the town of Buea, are covered by continuous tropical rainforests from lowland (often ~300 m a.s.l., although in some areas disturbed up to 700 m a.s.l.) to the timberline (~2,100–2,400 m a.s.l.), where the rainforest is replaced by montane and subalpine grasslands. Mount Cameroon is recognized as a hotspot of biodiversity and endemism for a wide range of taxa (Cronin, Libalah, Bergl, & Hearn, 2014), including Lepidoptera (Heppner, 1991; Maicher et al., 2016; Ustjuzhanin, Kovtunovich, Sáfián, Maicher, & Tropek, 2018; Yakovlev & Sáfián, 2016). The region is characterized by strong seasonality, mostly driven by the northward wet air movement during summer (monsoon), and the southward dry air movement from the Sahel during winter (harmattan) (Lefèvre, 1967). Annual precipitation usually exceeds 10,000 mm at the lower elevations of the southwestern slopes (making it one of the rainiest places in the world), with most of the rainfall concentrated between June and September, when monthly precipitation usually exceeds 1,500 mm (Lefèvre, 1967). Conversely, any rains between mid‐November and February are rare, especially at higher elevations. Two short transition seasons occur in March/May and October/November with a gradual increase and decrease of rainfall, respectively (Molua & Lambi, 2006).

All our material was sampled inside the Mount Cameroon National Park, at three sampling sites in the southwestern foothills of Mount Cameroon: around the Bamboo Camp (N 04.08990°, E 09.05174°; 350 m a.s.l.), the Drink Gari Camp (N 04.10221°, E 09.06304°; 650 m a.s.l.), and the PlanteCam Camp (N 04.11750°, E 09.07094°; 1,100 m a.s.l.). The first two sites are covered by a lowland rainforest with closed high canopy and relatively scarce understorey layers, while the forest around the PlanteCam Camp is already of an upland character, including a mixture of both lower and higher elevation forest elements. The latter locality is also relatively strongly affected by natural disturbances by forest elephants (*Loxodonta cyclotis*), reducing tree densities and creating forest openings dominated by various grasses, herbs, and ferns (Proctor et al., 2007). Such open areas were, however, avoided as much as possible during our sampling.

### 2.2 Lepidoptera sampling

All lepidopterans were sampled from 2014 to 2016, combining bait trapping and manual catching of specimens attracted by light. To cover most of the main seasonality aspects, lepidopterans were sampled in three different seasons: a transition from wet to dry seasons (November/December 2014), a high‐dry season (January/February 2016), and a transition from dry to wet seasons (April 2015). We did not sample during the high‐wet season as it would be impossible to keep the traps baited and the lights working during the heavy rains.

Within each of the three sites, fruit‐feeding lepidopterans were sampled in 16 circular plots placed in continuous forest or larger forest patches with a minimum distance of 150 m between each other (the same plots as in Ferenc et al., 2016). Within each plot (20 m radius), five Van Someren–Rydon type traps were exposed (modified IKEA PS Fångst hanging storage devices: height 75 cm, diameter 23 cm; first used by Sáfián, Csontos, & Winkler, 2011). Of these, four understory traps were installed as close to the ground as possible, and one canopy trap was set at a 20(±5) m height. Altogether, 80 traps were thus exposed at each site in each season. Each trap was baited by ca 0.3 L of fermented mashed bananas, refreshed daily, and completely replaced every three to five days according to bait condition. All the traps were exposed for 10 consecutive days within each sampling season. Every day, all captured butterfly and moth specimens were removed, killed, identified, and counted. Altogether, this study includes material collected by 240 traps, each exposed for 30 days (i.e., 7,200 “trap‐days” in total).

Within each of the three sites, moths were attracted by light in three plots selected to cover the main available forest habitats. These plots were placed at least a few 100 m distant from each other. In each of the three plots, the sampling was performed for two entire nights from dusk till dawn (6–7 p.m./a.m. depending on the season) per site and season, making 54 complete nights of light catching in total. Five nights before and after a full moon were avoided. Moths were attracted by an energy‐saving bulb (M036 produced by Hadex, Czechia: 4100 K, 5300 lm, 105 W, 230 V, 5U) placed in the center of two perpendicularly placed white sheets (1.5 × 1.5 × 1.8 m, the cloth type B produced by Entosphinx, Czechia). Each night, all individuals of the four focal moth groups (Arctiinae, Eupterotidae, Saturniidae, and Sphingidae) were caught by a jar saturated by vapors from an ammonia solution and stored for later identification.

For all analyses, we treated the six focal lepidopteran groups separately: bait‐trapped butterflies (mostly Satyrinae and Limenitidinae subfamilies of Nymphalidae, hereafter referred to as *butterflies*), bait‐trapped moths (mostly Erebidae, hereafter referred to as *fruit‐feeding moths*), and light‐attracted families Sphingidae, Saturniidae, and Eupterotidae, and the subfamily Arctiinae of Erebidae. Part of the material (most butterflies and some bait‐trapped moths, i.e., common Erebinae and Calpinae) was identified directly in the field; the rest was later mounted and identified into (morpho)species in a laboratory combining morphological features and genitalia dissections. Voucher specimens are stored in the Institute of Entomology, Biology Centre, Czech Academy of Sciences, České Budějovice, Czechia (bait‐trapped butterflies and moths), and the Nature Education Centre, Jagiellonian University, Kraków, Poland (all other focal groups, as well as a portion of the bait‐trapped species).

### 2.3 Species richness and diversity

All the following analyses were performed using the software R v. 3.4.3 (R core Team, 2017).

To estimate the completeness of the samples, individual‐based rarefaction curves of the species richness and sample coverage (i.e., the probability that a newly sampled individual would belong to the previously sampled species; Chao & Jost, 2012) curves were computed for each group in each season with the *iNEXT* package using 50 randomizations (Chao et al., 2014; Hsieh, Ma, & Chao, 2016). For an estimation of the total species richness of each focal group in each season, the bias‐corrected Chao1 species richness estimator was computed with the *SpadeR* package (Chao, Ma, Hsieh, & Chiu, 2016).

To avoid the known problems with incomplete inventories and to allow better comparability with other studies, our interseasonal biodiversity comparisons were based on four different metrics, all based on the critical review by Beck and Schwanghart (2010). To compare communities, we used the following indices: (a) *abundance*, that is, the number of sampled individuals; (b) *species richness*, that is, the number of recorded species; (c) the *bias‐controlled effective number of species* (eHbc) based on bias‐corrected Shannon's entropy, currently considered as one of the most suitable measures of biodiversity in potentially undersampled communities (Beck & Schwanghart, 2010), and (d) *Fisher's α*, the diversity index often used in entomological studies of biodiversity for its relative independence on sample size and robustness for comparisons of incomplete inventories. The latter two indices were computed using the *entropart* (Marcon & Hérault, 2015) and *vegan* (Oksanen et al., 2017) packages, respectively.

To test the interseasonal differences in all four measures, the generalized linear mixed‐effect models (GLMM) were applied, with *season* as a fixed factor, and *sites* and *plots* (nested in *sites*) as random‐effect variables. Each sample was comprised of all specimens collected within each plot in the 10 sampling days for the bait‐trapped material (i.e., all five traps and 10 days of bait trapping per plot were pooled to form a sample), and within each plot in two sampling nights for the light‐attracted material (i.e., the two sampling nights per plot were pooled to form a sample). Species richness and abundance were fit into the models with negative binomial distribution (O'Hara & Kotze, 2010), and eHbc and Fisher's *α* were log‐transformed in order to improve the parametric test assumptions, based on the models' residuals. The pairwise post hoc comparisons of the least square means with Tukey adjustment were then applied among the particular sampled seasons. All models were computed using the *lme4 *package (Bates, Mächler, Bolker, & Walker, 2015). To quantify the proportion of variance explained by seasonality after excluding the effects of the random factors, we have also computed its marginal *R* ^2^ from all significant models using the *piecewiseSEM* package (Lefcheck, 2016).

### 2.4 Species turnover

To quantify the interseasonal changes in species composition, we used measures of beta‐diversity. Beta‐diversity was partitioned into two additive components: (a) interseasonal *species turnover* and (b) *nestedness *of communities occurring in individual seasons (Baselga, 2010, 2012 ). The first represents the part of the total dissimilarity caused by species turnover among individual seasons. The latter represents the part of the total dissimilarity caused by the fact that the species‐poorer community is a subset of the richer one. For each group, the incidence‐based Sørensen dissimilarity index (*β*_sør_; Baselga, 2010) was used as an estimation of the total dissimilarity between all pairwise combinations of the seasons. *β*_sør_ was then partitioned into the Simpson dissimilarity index (*β*_sim_), reflecting the dissimilarity caused by the species turnover, and into the nestedness (*β*_nes_), reflecting the dissimilarity caused by the communities' nestedness. All the indices were computed with the *betapart *package (Baselga & Orme, 2012)*.*

### 2.5 Community composition

Interseasonal changes in species composition of the sampled communities were analyzed by multivariate ordination methods (Šmilauer & Lepš, 2014). For all analyses, material from all five traps per plot and 10 sampling days per season was pooled. To reveal if individual samples (plots) cluster mainly according to the sampling season, Nonmetric Multidimensional Scaling analyses (NMDS) with Bray–Curtis dissimilarity matrices were run for each focal group separately. Because NMDSs revealed a strong influence of the sampling sites on the communities' composition of all focal groups, the influence of season was tested by partial canonical correspondence analyses (CCA) with *season* as the explanatory variable and *site* as the covariate. The log‐transformed (*n* + 1) abundances of individual Lepidoptera species were used as the response variables (Šmilauer & Lepš, 2014). All ordination analyses were tested by Monte Carlo permutation tests with 999 permutations. All ordination analyses were performed in Canoco 5 (ter Braak & Šmilauer, 2012).

## 3 RESULTS

### 3.1 Total species richness and abundance

In total, 20,576 individuals of all focal groups were collected. From these numbers, 16,062 individuals (10,425 butterflies, 5,637 fruit‐feeding moths) belonging to 403 (morpho)species (117 butterflies, 286 fruit‐feeding moths) were bait‐trapped, and 4,514 individuals (3,645 Arctiinae, 517 Sphingidae, 252 Saturniidae, and 100 Eupterotidae) of 156 (morpho)species (86 Arctiinae, 38 Sphingidae, 15 Saturniidae, and 17 Eupterotidae) of the focal moth groups were attracted by light (Table 1).

**Table 1:** Summary of abundance and diversity of individual focal groups of Lepidoptera in different seasons on Mount Cameroon

Focal group	Season	Total abundance	Total number of species	eHbc^a^	Fisher's *α*	Chao1 (±*SE* b)	SC^c^
Butterflies	Wet to dry	1,701	88	**37.57**	**19.68**	99.7 (±7.9)	**0.99**
	Dry	**6,789**	**101**	36.33	16.83	**106.6 (±5.3)**	**0.99**
	Dry to wet	1,935	88	33.94	18.99	102.2 (±8.6)	**0.99**
	Total	10,425	117	44.1	18.46	130.0 (±9.3)	0.99
Fruit‐feeding moths	Wet to dry	1,238	146	42.54	43.02	239.1 (±33.3)	0.95
	Dry	1,841	152	**44.74**	39.29	203.3 (±18.1)	**0.97**
	Dry to wet	**2,558**	**186**	43.89	**46.11**	**267.3 (±26.5)**	**0.97**
	Total	5,637	286	57.9	63.62	443.5 (±42.8)	0.98
Arctiinae	Wet to dry	845	60	27.35	14.76	63.0 (±2.9)	**0.99**
	Dry	1,248	62	20.62	13.71	75.1 (±9.0)	**0.99**
	Dry to wet	**1,552**	**79**	**31.92**	**17.59**	**91.0 (±7.9)**	**0.99**
	Total	3,645	86	32.8	15.79	102.5 (±12.9)	0.99
Sphingidae	Wet to dry	111	16	6.2	5.13	25.0 (±8.0)	0.92
	Dry	**262**	**24**	**7.12**	**6.43**	**36.0 (±10.7)**	**0.96**
	Dry to wet	144	20	5.26	6.31	33.6 (±11.1)	0.92
	Total	517	38	7.09	9.45	60.62 (±14.9)	0.97
Saturniidae	Wet to dry	40	7	5.09	2.46	8.0 (±2.2)	0.95
	Dry	**132**	**11**	2.95	2.85	14.0 (±4.1)	**0.97**
	Dry to wet	80	**11**	**7.29**	**3.45**	**20.9 (±10.1)**	0.94
	Total	252	15	7.14	3.49	20.0 (±6.0)	0.98
Eupterotidae	Wet to dry	**54**	**14**	11.87	6.13	**15.5 (±2.2)**	**0.93**
	Dry	15	9	**13.35**	**9.5**	13.7 (±5.2)	0.62
	Dry to wet	31	10	9.7	5.12	12.9 (±4.1)	0.87
	Total	100	17	12.5	5.88	20.0 (±4.1)	0.96

Note

The highest values of each diversity measure for each focal group are indicated in bold.

a The bias‐controlled effective number of species based on bias‐corrected Shannon's entropy.

b Standard error.

c Sampling coverage.

For both bait‐trapped taxa, the individual‐based rarefaction curves closely approach the asymptote, indicating relatively well‐sampled communities, especially concerning butterflies (Supporting Information Figure S1–S3). Concerning the light‐attracted moth groups, the individual‐based rarefaction curves and Chao1 estimators suggested relatively lower sampling coverage (Supporting Information Figure S1–S3, Table 1). However, the sample coverages of all individual groups in each season are generally well over 90%, indicating well‐sampled communities (Supporting Information Figure S1–S3, Table 1), except for Eupterotidae with relatively undersampled communities during the high‐dry season and the transition from dry to wet seasons.

**Figure 2:**
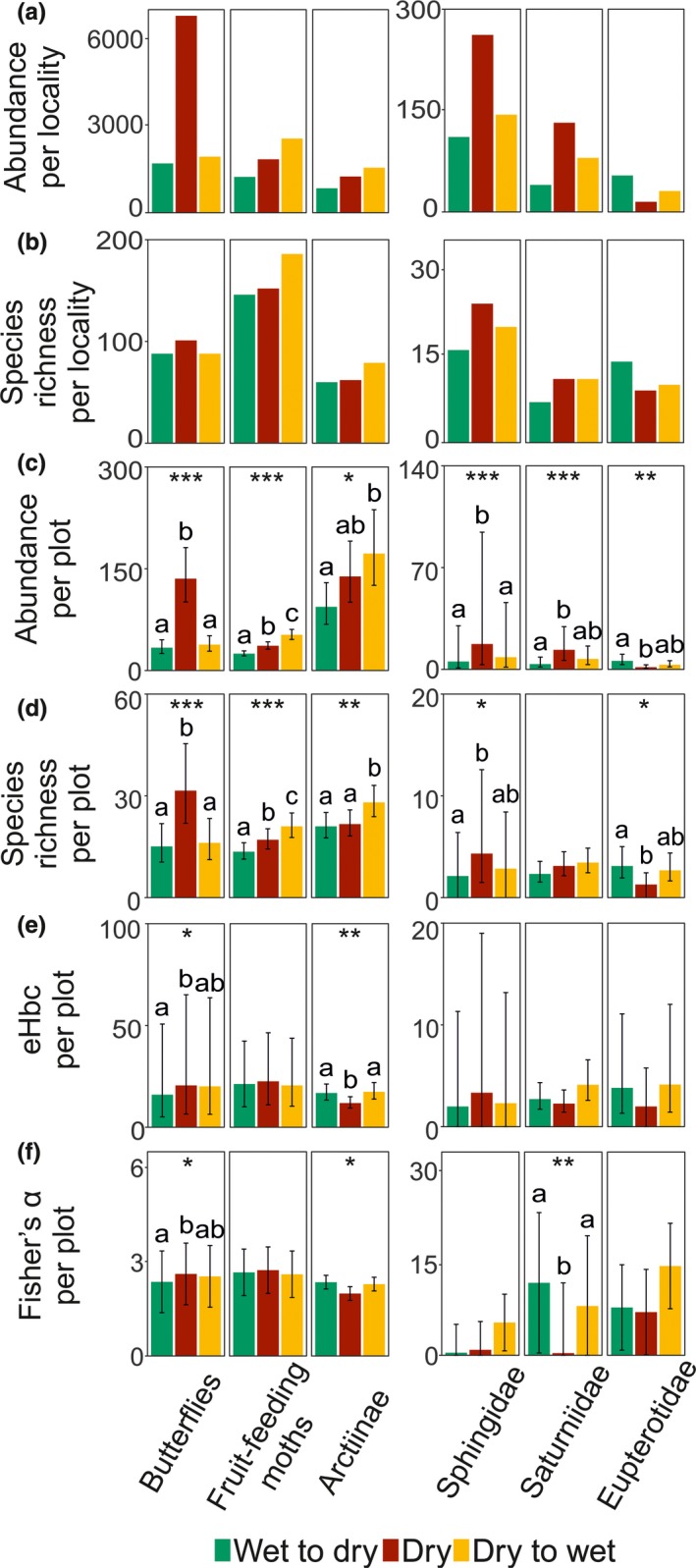
Mean (a) abundance and (b) species richness per locality during distinct sampling seasons. Results of GLMMs of (c) abundance, (d) species richness, (e) bias‐controlled effective number of species, and (f) Fisher's *α* per sampling plot, sampled by standardized bait trapping (butterflies and fruit‐feeding moths) and light attraction (Arctiinae, Eupterotidae, Sphingidae, and Saturniidae). Means per plot with 95% unconditional confidence intervals are visualized. The GLMM results of individual models are included (the type II Wald *χ* ^2^ tests: *p < 0.05; **p < 0.01; ***p < 0.001); see Table 2 for more detailed results

Both total abundance and species richness per site were lowest during the transition from wet to dry seasons for all focal groups, except Eupterotidae for whom total abundance was lower during the high‐dry season (Table 1, Figure 2). Total abundance was highest in the high‐dry season for butterflies, Sphingidae and Saturniidae, and in the transition from dry to wet seasons for fruit‐feeding moths and Arctiinae. Eupterotidae were most abundant during the transition from wet to dry seasons (Table 1, Figure 2). The Chao1 followed the same patterns as total species richness for all groups, except Saturniidae with the highest Chao1 in the transition from wet to dry seasons (Table 1). The total eHbc showed different patterns (Table 1). It was highest either in the high‐dry season (for fruit‐feeding moths, Sphingidae and Eupterotidae), in the transition from wet to dry seasons (for butterflies), and in the transition from dry to wet seasons (for Arctiinae and Saturniidae). Fisher's *α* followed a similar pattern, except for fruit‐feeding moths with the highest value in the transition from dry to wet seasons.

### 3.2 Local species richness and diversity

The GLMMs of abundance and species richness per sampling plot (Figure 2, Table 2) revealed the high‐dry season communities significantly most abundant and richest for butterflies and Sphingidae, and poorest for Eupterotidae. Fruit‐feeding moths and Arctiinae were shown to be significantly richest in individual plots in the transition from dry to wet seasons. Saturniidae did not reveal any significant interseasonal pattern in species richness per plot but were most abundant in the high‐dry season. Similarly, the high‐dry season eHbc per plot (Figure 2) was significantly highest for butterflies and significantly lowest for Arctiinae. The other biodiversity models did not show any significant effects of seasonality on the diversity of the individual focal groups. Seasonality was revealed also as the crucial factor influencing abundance of the studied groups, as the proportion of its explained conditional variability exceeded 39% for all of them, except Sphingidae for whom it explained 10% of the variability. The proportion of variability in species richness explained by seasonality was lower, but still exceeded 26% for all the significant models, except for Sphingidae again with 8% of the explained variability (Table 2).

**Table 2:** Summaries of the GLMMs results for individual models

Focal group	Response variable	*χ* ^2^	*df*	*p*‐Value	Marginal *R* ^2^
Butterflies	Abundance	364.1	2	<0.01	0.79
	Species richness	289.59	2	<0.01	0.43
	eHbc	8.42	2	0.01	0.02
	Fisher's *α*	6.99	2	0.03	0.02
Fruit‐feeding moths	Abundance	61.33	2	<0.01	0.59
	Species richness	40.07	2	<0.01	0.29
	eHbc	0.72	2	0.70	—
	Fisher's *α*	1.12	2	0.57	—
Arctiinae	Abundance	7.11	2	0.03	0.90
	Species richness	11.63	2	<0.01	0.26
	eHbc	9.71	2	<0.01	0.26
	Fisher's *α*	6.71	2	0.03	0.20
Sphingidae	Abundance	13.9	2	<0.01	0.10
	Species richness	8.74	2	0.01	0.08
	eHbc	4.81	2	0.09	—
	Fisher's *α*	5.20	2	0.07	—
Saturniidae	Abundance	14.43	2	<0.01	0.39
	Species richness	2.04	2	0.36	—
	eHbc	4.23	2	0.12	—
	Fisher's *α*	11.38	2	<0.01	0.22
Eupterotidae	Abundance	12.45	2	<0.01	0.47
	Species richness	6.99	2	0.03	0.27
	eHbc	4.58	2	0.10	—
	Fisher's *α*	3.04	2	0.22	—

### 3.3 Beta diversity

The pairwise Sørensen total dissimilarities varied greatly among taxa (Table 3). The communities of fruit‐feeding moths, Sphingidae, Saturniidae, and Eupterotidae, were shown as the most dissimilar among the seasons (*β*_sør_ ranging from 0.25 and 0.61). The beta‐partitioning of the total dissimilarity revealed that the majority of the total dissimilarity among the sampled seasons can be explained by species turnover (more than 80% of *β*_sør _for all season combinations) for fruit‐feeding moths and Sphingidae. For Saturniidae and Eupterotidae, the total dissimilarity between the two transition seasons was mostly explained by the nestedness in the transition from dry to wet seasons for Saturniidae (57% of *β*_sør_), while Eupterotidae revealed the opposite pattern (60% of *β*_sør_).

**Table 3:** Partitioning of beta‐diversity among the sampled seasons and for individual focal groups of Lepidoptera into nestedness and species turnover

Total dissimilarity and nestedness		
Butterflies		
Dry to wet (88)	0.18 (32%)	
Wet to dry (88)	0.16 (37%)	0.15 (0%)
	Dry (101)	Dry to wet (88)
Fruit‐feeding moths		
Dry to wet (186)	0.46 (13%)	
Wet to dry (146)	0.44 (2%)	0.44 (17%)
	Dry (152)	Dry to wet (58)
Arctiinae		
Dry to wet (79)	0.19 (**58%**)	
Wet to dry (60)	0.16 (6%)	0.19 (**66%**)
	Dry (62)	Dry to wet (79)
Sphingidae		
Dry to wet (20)	**0.50** (10%)	
Wet to dry (16)	**0.55** (20%)	**0.61** (8%)
	Dry (24)	Dry to wet (20)
Saturniidae		
Dry to wet (11)	0.36 (0%)	
Wet to dry (7)	0.44 (36%)	0.33 (**57%**)
	Dry (11)	Dry to wet (11)
Eupterotidae		
Dry to wet (10)	0.47 (6%)	
Wet to dry (14)	0.39 (43%)	0.25 (**60%**)
	Dry (9)	Dry to wet (10)

Note

The values represent the pairwise Sørensen dissimilarity indices (in bold if >0.50). The proportions in parentheses represent the part of total dissimilarity caused by the nestedness (in bold if >50%), while the remaining part represents the species turnover. The numbers in parentheses behind the seasons stand for the number of collected species.

The communities of butterflies and Arctiinae were relatively more similar among the sampled seasons (*β*_sør_ ranging from 0.15 and 0.19). This dissimilarity was mainly explained by the species turnover, especially between the transition from wet to dry seasons and the high‐dry season for Arctiinae (94%), and between the two transition seasons for butterflies (100%).

### 3.4 Species composition

**Figure 3:**
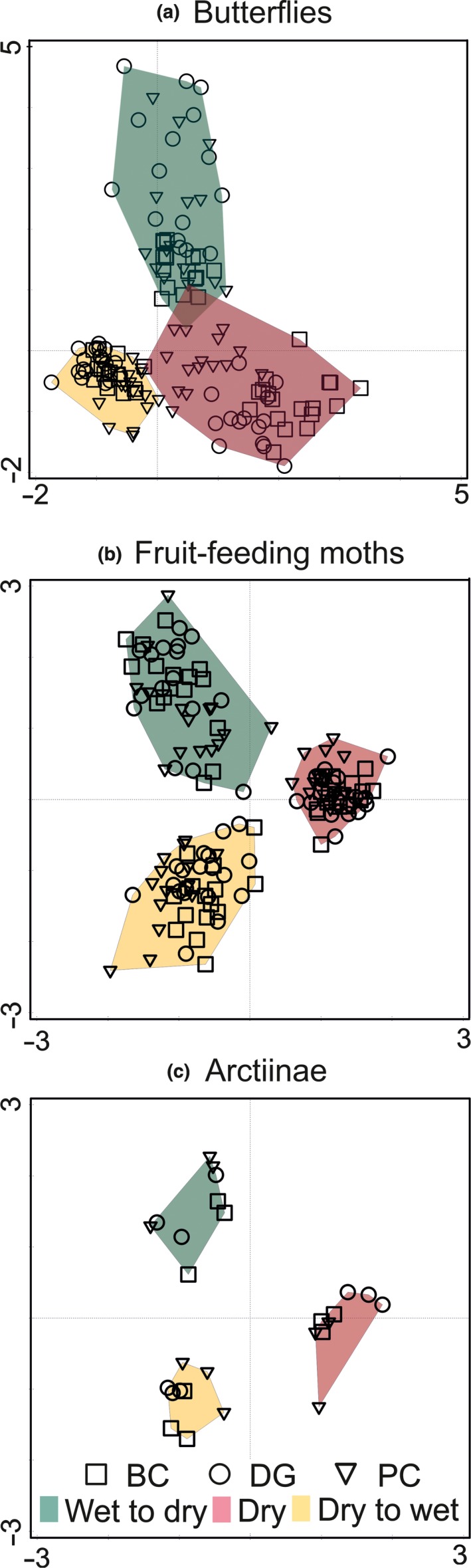
Ordination diagrams of the partial CCA with *season* as the explanatory variable, and *site* as the covariate. Individual samples from different seasons (distinguished by polygons of different colors) and localities (distinguished by different symbols) are visualized. Only the three focal groups with the significant influence of season are shown: (a) butterflies, (b) fruit‐feeding moths, and (c) Arctiinae. See Table 4 for all analyses results

The partial CCAs (Figure 3, Table 4) revealed significant interseasonal differences in the community composition for butterflies, fruit‐feeding moths, and Arctiinae, with relatively well‐separated communities of all three sampled seasons (although a small overlap between both transitions was detected for butterflies). No significant interseasonal differences in the community composition were revealed for Sphingidae, Saturniidae, and Eupterotidae.

**Table 4:** Summary of the partial CCAs exploring the effect of seasons on community composition for individual Lepidoptera groups

	All axes eigenvalues	Explained variation (%)	Pseudo‐*F*	*p*‐Value
Butterflies	1.73	12.1	10.7	0.005
Fruit‐feeding moths	3.79	8.5	7.6	0.005
Arctiinae	1.18	15.5	3.2	0.001
Sphingidae	2.03	4.9	1.5	1
Saturniidae	2.21	14.3	3.0	0.5
Eupterotidae	3.47	0.7	1.1	1

Note

The numbers show the eigenvalues accounted for all axes, as well as the adjusted variation explained by the effect of seasonality. The pseudo‐*F* statistics and *p*‐values were obtained by Monte Carlo tests with 999 permutations.

## 4 DISCUSSION

We identified seasonality as a crucial factor for forming adult communities of Lepidoptera in the studied West African tropical rainforest, although the phenological patterns slightly differed among the particular groups studied. Both species richness and abundance were generally lower at the end of the wet season and increased toward the high‐dry season. Whereas we do not have any data from the high‐wet season itself, the low abundance and diversity of adult Lepidoptera can be related to the climatic harshness of the high‐wet season on Mount Cameroon (one of the wettest places in the world, see above). These patterns corroborate with several studies of sphingids (Cruz‐Neto et al., 2011; Owen, 1969) and butterflies (Aduse‐Poku et al., 2012; DeVries et al., 2012; Grøtan et al., 2014, 2012 ; Ribeiro et al., 2010) from other tropical areas. Richer and more abundant communities during wet seasons are known for Neotropical fruit‐feeding butterflies (Checa et al., 2014; DeVries & Walla, 2001; DeVries et al., 1997). These studies, however, did not originate from areas with such strong seasonality and extreme wet season.

The high abundance and diversity of adult tropical lepidopterans in the dry season are often interpreted by requirements of their adult and larval stages. During the wet season, adults have less time for various activities including feeding, mating, and dispersal behavior, particularly in sun‐dependent butterflies. Although there are no studies of vegetation phenology on Mount Cameroon, the flowering peak of many abundant trees, representing the main source of nectar in the local communities, seems to be during the high‐dry season according to our experience. But the high humidity and strong precipitation can also affect caterpillars both negatively (such as higher activity of pathogens, higher predation rate, or mechanical disturbance by strong rains; Janzen, 1993; Intachat et al., 2001; Hill et al., 2003; Molleman et al., 2016) and positively (by common mass sprouting of young leaves of various host plants). In the extreme wet season on Mount Cameroon, we hypothesize that negative effects dominate, as the extremely low solar radiation negatively influences foodplants' photosynthesis and high water stress decreases production of new plant tissues (van Schaik, Terborgh, & Wright, 1993; Wright, 1996). Although no exact data exists, we experienced the main vegetation sprouting in the transition from dry to wet season, while in the beginning of the dry season many herbs are also growing and flowering. Considering the dramatic rainfall discrepancy between the high‐dry and high‐wet seasons on Mount Cameroon, we hypothesize that the highest abundance and diversity of most studied lepidopteran groups during the transition from dry to wet seasons reflect a suitable compromise for adults and resprouting of vegetation before the heavy rains. Altogether, the adults' activity seems to be favorable during the high or late dry season, allowing concentration of the main abundance of caterpillars into the beginning of the wet season in the studied area. On the other hand, we do not have any data on caterpillar activity or abundance to support this hypothesis. Simultaneously, we cannot dismiss the biannual adult lepidopterans pattern with its second peak in the high‐wet season known from some other studies (Checa et al., 2014; DeVries et al., 1997; Devries & Walla, 2001) as we do not have any data from this period. However, considering the extreme local rainfalls during the high‐wet season, such a pattern is not very probable.

Our interpretation of the seasonal patterns of abundance and species richness of adult Lepidoptera can be also seen through particular differences among the groups: both measures increased for fruit‐feeding moths and Arctiinae, and decreased for all other groups but Eupterotidae during the transition from dry to wet seasons. A simple explanation can be proposed for fruit‐feeding butterflies, the only focal group with day‐time activity. Their adults strongly depend on sunshine, decreasing toward the high‐wet season. The potential artifact of thirsty adult butterflies entering the baited traps in search for water during the high‐dry season (Freitas et al., 2014) can be doubted by the different pattern observed for fruit‐feeding moths. Nevertheless, such different biodiversity patterns of fruit‐feeding butterflies and moths, belonging to the same trophic guild, were unexpected. Although the peak of fruit‐feeding butterflies' biodiversity during the dry season has already been repeatedly documented (Aduse‐Poku et al., 2012; DeVries et al., 2012; Grøtan et al., 2014, 2012 ; Ribeiro et al., 2010), no comparable study on fruit‐feeding moths exists to our knowledge. As we know from our observations, many fruit‐feeding moth taxa (e.g., *Deinypena, Hesperochroa, Pseudoarcte*) are only moderately attracted to artificial light; it is thus impossible to speculate on these patterns by comparing them with light‐attracted moths. Yet, it can be hypothesized that the differences are driven by the different use of resources by the two groups. A substantial part of the recorded butterflies was composed of relatively large and mobile species (e.g., *Charaxes*, *Euphaedra*, *Cymothoe*) with potentially high demands for energy, while the recorded communities of fruit‐feeding moths were mostly composed of smaller species with lower energetic demands on average (Niven & Scharlemann, 2005). However, during the wet season, including its beginning, there are abundant fleshy fruits on the ground (pers. observ.), resembling studies from Ghana (Lieberman, 1982) and Rwanda (Sun et al., 1996). Nevertheless, Adamescu et al. (2018) demonstrated both the same and different patterns in different Afrotropical forest communities, and without any local quantitative data, we rather avoid any generalizations. Nevertheless, we hypothesize that the differing biodiversity patterns reflect differences in dependency on direct sunshine among the two fruit‐feeding groups. It can be also hypothesized that these different biodiversity peaks could be caused by seasonal food niche partitioning, but no studies on tropical Lepidoptera are yet related to this topic.

The other two focal taxa with feeding adults also differed in their biodiversity patterns during the transition from dry to wet seasons: abundance and species richness increased for Arctiinae, while decreased for Sphingidae. Because most Arctiinae in our material were lichen moths (Lithosiini) with well‐developed proboscides and probably feeding on various sugar resources similarly to the fruit‐feeding moths (although some minor arctiin groups include nonfeeding adults, Schulze, Linsenmair, & Fiedler, 2001), we offer similar explanations as discussed above. The only quantitative study on this group in the tropics revealed no specific biodiversity pattern related to seasonality in southern Ecuador (Hilt et al., 2007). In contrast, the dry season biodiversity and abundance peaks of Sphingidae were well documented in different tropical areas, usually interpreted in relation to a synchronicity with flowering of plants with specialized flowers and consequent saturation of local communities by dry season vagrants (e.g., Owen, 1969; Cruz‐Neto et al., 2011). On Mount Cameroon, no proper dataset on the flowering phenology exists. However, we observed flowering peaks of individual sphingophilous plants (e.g., *Ixora, Schumanophyton, Tabernaemontana*) during the dry season. Simultaneously, several vagrant hawkmoths (e.g., *Nephele aequivalens* (Walker, 1856), *Phylloxiphia bicolor *(Rothschild, 1894), *Pierreclanis admatha* (Pierre, 1985) were detected during the high‐dry season only. We thus hypothesize that flowering of these specialized plants might, at least partially, explain the observed patterns.

The two focal taxa with nonfeeding adults, Saturniidae and Eupterotidae, did not show any consistent seasonal patterns of biodiversity nor abundance. Despite the lack of comparative studies, this could be related to their short‐living adults and the related strong temporal species turnover, as shown in our study as well. Simultaneously, a lower seasonal stress can be expected for the short‐living adults; their phenology can thus be driven by different mechanisms than for the other Lepidoptera groups.

For all taxa, we also revealed a strong effect of seasonality on their community compositions, caused mainly by the strong interseasonal species turnover. The fruit‐feeding Lepidoptera and Arctiinae showed distinct phenological guilds in all three sampled seasons, indicating a strong seasonal specialization of communities. For fruit‐feeding butterflies, this is consistent with long‐term studies conducted in other tropical regions (Grøtan et al., 2014, 2012 ; Valtonen et al., 2013), while no similar studies exist for fruit‐feeding moths. The distinct seasonality of Arctiinae contradicts with Hilt et al. (2007), who reported many tropical Arctiinae occurring all year‐long. The communities of Sphingidae, Saturniidae, and Eupterotidae showed relatively high interseasonal dissimilarities, but no significant differences in community composition patterns. We consider this as an artifact of the relatively low species richness together with the presence of several abundant phenological generalists in all these groups (e.g., Sphingidae: *Polyptychus nigriplaga* Rothschild & Jordan, 1903; Saturniidae: *Imbrasia epimethea* (Drury, 1773); Eupterotidae: *Stenoglene *sp.). Concerning Sphingidae, previous studies revealed no distinct seasonality of their communities as well (Beck & Linsenmair, 2006; Owen, 1969). We do not know any similar study for the other two groups. Contrastingly, we found a relatively large proportion of Sphingidae and Saturniidae to be specialized for the high‐dry and transition from dry to wet seasons on Mount Cameroon.

The strong interseasonal patterns found by our study can indicate a high sensitivity of the local communities to the expected consequences of the global change. The annual variability of the precipitation timing, length, and magnitude has been increasing in the tropics over the past decades (Feng et al., 2013). The combination of the changing climatic conditions and consequent shifts in host plants' phenology (Cleland, Chuine, Menzel, Mooney, & Schwartz, 2007) could cause serious, but hardly predictable, changes on seasonally specialized Lepidoptera communities in the Mount Cameroon area. Considering that Lepidoptera play key roles in all their developmental stages as primary consumers, pollinators and prey, such expected changes of their seasonal patterns might affect entire ecosystems through both top‐down and bottom‐up effects.

## CONFLICT OF INTEREST

None declared.

## AUTHORS CONTRIBUTION

R.T., Sz.S., and V.M. conceived and designed the study; V.M., Sz.S., M.M., E.B.F., S.J., and R.T. collected the material; V.M., Sz.S., M.M., L.P., T.P., and R.T. processed and identified the material; V.M. and R.T. analyzed the data, interpreted the results, and wrote the first manuscript draft; and all authors provided critical feedback and helped shape the final manuscript.

## DATA ACCESSIBILITY

Data available from the Dryad Digital Repository: https://doi.org/10.5061/dryad.sc1dr77.
