# Supplementary material for: Flying between raindrops: Strong seasonal turnover of several Lepidoptera groups in lowland rainforests of Mount Cameroon
Source: Ecol Evol. 2018 Dec 3;8(24):12761–72. doi: 10.1002/ece3.4704 (PMC6308855; doi:10.1002/ece3.4704)
Supplement: Supplementary file 1 [file ECE3-8-12761-s001.docx]

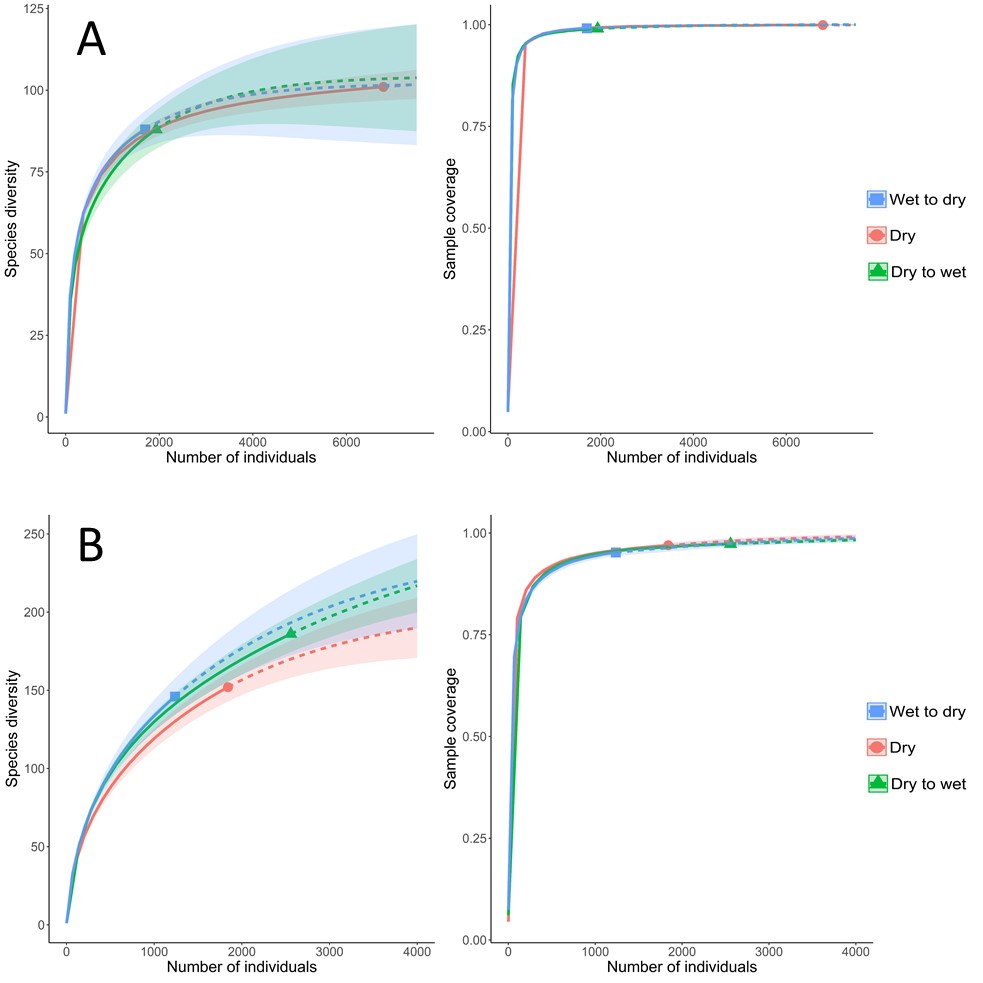


Figure S1. Individual-based rarefaction curves of species richness (q=0) of (A) butterflies and (B) fruit-feeding moths recorded in particular sampled seasons with 95% confidence intervals are represented on the left. Coverage-based sampling curves with 95% confidence intervals are represented on the right. The solid lines represent the rarefied reference samples, while the dashed lines represent the extrapolated samples.


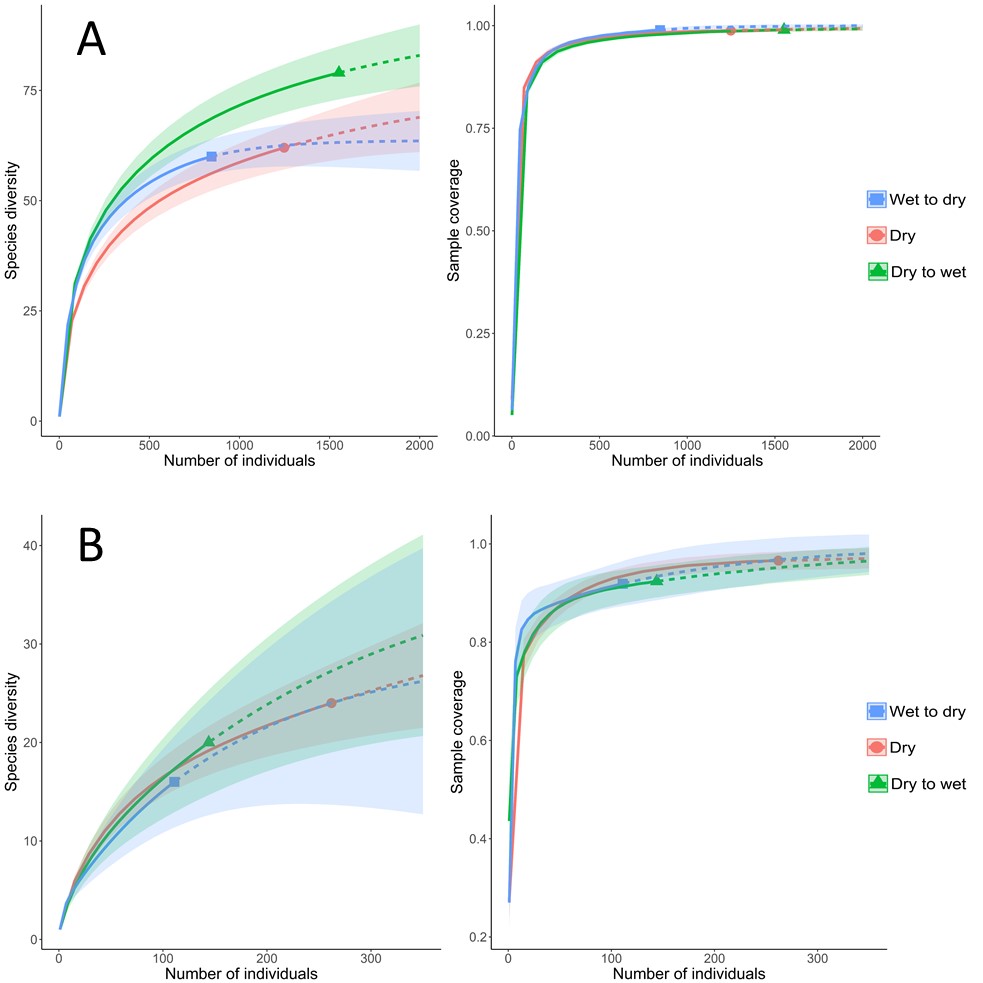


Figure S2. Individual-based rarefaction curves of species richness (q=0) of (A) Arctiinae and (B) Sphingidae recorded in particular sampled seasons with 95% confidence intervals are represented on the left. Coverage-based sampling curves with 95% confidence intervals are represented on the right. The solid lines represent the rarefied reference samples, while the dashed lines represent the extrapolated samples.


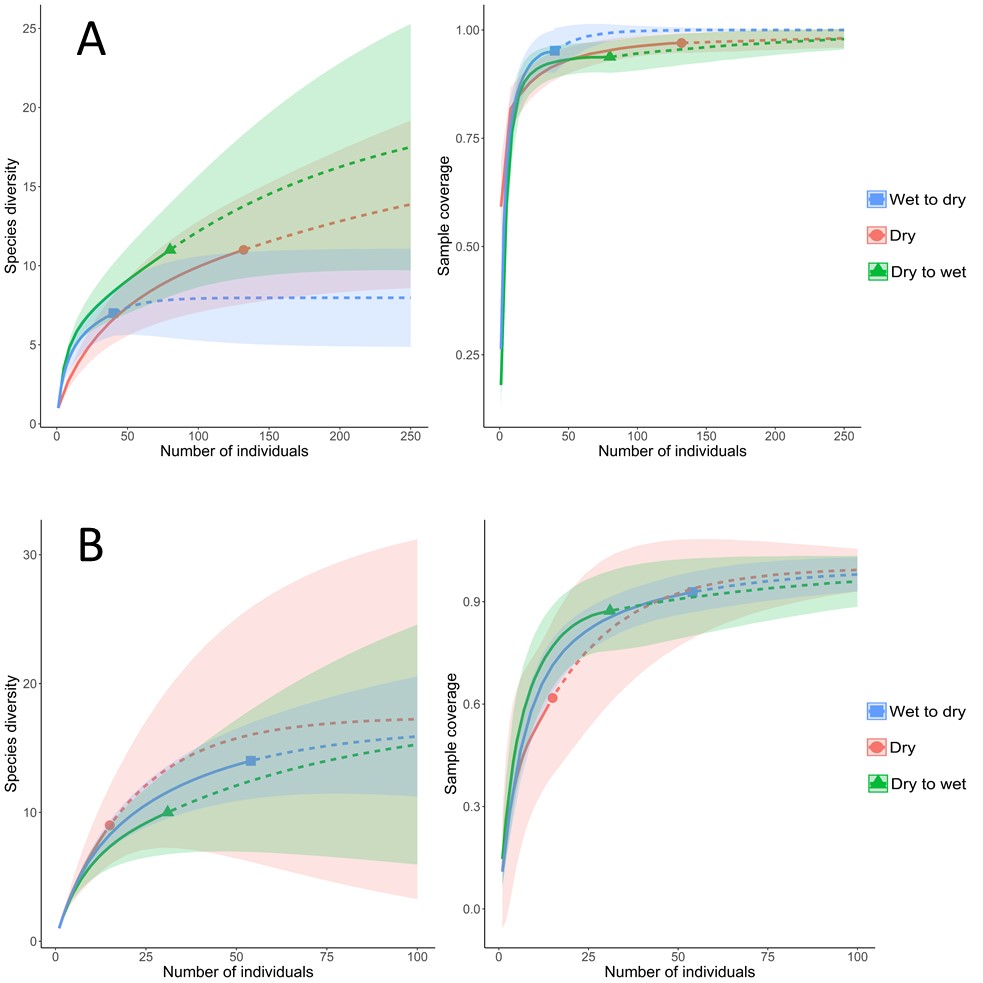


Figure S3. There are individual-based rarefaction curves of species richness (q=0) of (A) Saturniidae and (B) Eupterotidae collected in particular sampled seasons with 95% confidence intervals are represented on the left. Coverage-based sampling curve with 95% confidence intervals are represented on the left. The solid lines represent the rarefied reference samples, while the dashed lines represent the extrapolated samples.
